# Overweight and obesity among children under five in Ethiopia: further analysis of 2016 national demographic health survey: a case control study

**DOI:** 10.1186/s13104-019-4752-8

**Published:** 2019-10-31

**Authors:** Haftom Gebrehiwot Weldearegay, Tesfay Gebregzabher Gebrehiwot, Mulugeta Woldu Abrha, Afework Mulugeta

**Affiliations:** 1 College of Health Sciences, Mekelle University, Mekelle, Ethiopia; 2 Tigray Health Research Institute, Mekelle, Ethiopia

**Keywords:** Overweight and obesity, EDHS, Case–control study, Ethiopia

## Abstract

**Objective:**

The objective of this study was to assess the determinants of overweight and obesity among children under 5 years in Ethiopia.

**Results:**

Data from a total of 672 (224 cases and 448 controls) under 5 years of age children were included in the study. Urban residence (AOR = 2.63, 95% CI 1.29, 5.34), boys (AOR = 1.56, 95% CI 1.10, 2.22) and age of the child less than 6 months (AOR = 3.40, 95% CI 2.05, 5.64) were the determinants for being childhood overweight and obesity.

## Introduction

Overweight and obesity are defined as abnormal or excessive accumulation of fat which may impair health [1, 2]. A combination of childhood overweight and obesity are of the most serious public health challenges of the twenty first century [2]. The prevalence of childhood overweight and obesity is increasing in all countries, with the most rapid rise in low and middle-income countries with majority of overweight and obese children live in developing countries, where the rate of increase has been more than 30% higher than that of developed countries [3–5].

In Africa, the number of overweight and obese children has nearly doubled from 5.4 million in 1990 to 10.3 million in 2014 [6]. The occurrence of infant, childhood and adolescent overweight and obesity may be plateauing in African settings, but in absolute numbers there are more overweight and obese children living in low and middle-income countries compared to in high-income countries [5, 7].

Moreover, children with overweight and obesity suffer severe health consequences in childhood and are at high risk of becoming obese adults, resulting increased risk of non-communicable diseases and reproductive disorders later in their life [1, 4]. Many factors can be linked with overweight and obesity in children. Economic growth or socioeconomic status, maternal level of education, marital status, smoking during pregnancy, sex of the child, birth weight and the child’s birth rank, area of residence, location of residence, age of the child, BMI of parents have been found as risk factors of childhood overweight and obesity [5, 8–10].

In Ethiopia, a low-income country which is still scarred by childhood malnutrition, childhood obesity is not yet perceived as an emerging health issue and receives little attention. According to UNICEF 2017 annual report there is overall increment of prevalence of overweight among children from 1.7 to 3.6% in Ethiopia [11]. Therefore, within this evidence, there are incongruent results in the determinant factors of overweight and obesity among literatures in Ethiopia. This study aimed to identify the determinants of overweight and obesity among children in Ethiopia. Thus, this finding highlighted in designing effective preventive strategies to clench the rising burden of early childhood overweight and obesity and its consequential morbidity and mortality in adulthood.

## Main texts

### Methods and materials

#### Data source

We used a secondary data analysis from 2016 Ethiopia Demographic and Health Survey. It is the fourth DHS conducted in Ethiopia by the Central Statistical Agency (CSA) through the request of Federal Ministry of Health (FMoH) in collaboration with United States Agency for International Development.

#### Sampling techniques and population

We used data from the 2016 Ethiopian Demographic Health Survey (EDHS) assessment. The EDHS was a national representative sample of 18,008 households and 10,752 children under age 5 years were eligible for height and weight measurements [12]. The ‘child record’ (age ranged from 0 to 59 months) data set was downloaded from the MEASURE DHS website. Child and maternal anthropometric data and various pertinent socio demographic variables were extracted from the data sets.

The nutritional status of children was categorized into normal weight (above minus two standard deviations (− 2 SD) and below plus two standard deviations (+ 2 SD) from the median of the reference population), underweight (below minus two standard deviations (− 2 SD) from the median of the reference population) and overweight and obese (more than two standard deviations (+ 2 SD) above the median of the reference population) based on the BMI Z scores.

We retrieved children with underweight and those whose BMI z-scores were missing or was recorded as “Height out of plausible limits” or “Age in days out of plausible limits” or “Flagged cases”, as their values were unusable since they were recorded in the database under special codes which corresponded either to responses that were considered inconsistent with other response in the questionnaire and thought to be probably an error, or to responses whose value was “Don’t know”. All eligible overweight and obese children (n = 224) were included into the analysis as cases. Similarly, 448 normal weight children were selected randomly as controls from 7168 normal weight children living with their mothers and incorporated into the further analysis.

Outcome variable: Children who had BMI z-score over 2 were classified as overweight and obese children characterized as cases. And those who had BMI z-score ranged from − 2 to 2 were classified as normal weight children, which are considered as controls [13].

#### Data analysis

Data analyses were carried out using SPSS 21 ™—software. Variables with p < 0.25 at bivariate analysis were entered to multivariable logistic regression analysis using the forward likelihood ratio method. Finally, p < 0.01 in multivariable analysis was considered to declare statistically significant association between predictors and outcome of interest.

### Results

#### Socio-demographic characteristics of under five children

We enrolled 224 cases and 448 controls of children aged 0–59 months old. Mean age of cases was 23.6 (SD ± 16.3) months and mean age of controls was 32.3 (SD ± 16.6) months. Two-third, 150 (67.0%) of the cases and majority 382 (85.3%) of the controls were from rural areas. Nearly one-fifth 46 (20.5%) of the cases and 71 (15.8%) controls were from Oromia region.

Sixty-two percent (n = 141) of cases belonged to the low birth rank (1–3 births) and almost half 230 (51.3%) of controls belonged to high birth rank category (> 3 births) (Table 1).

**Table 1:** Socio-demographic characteristics of under five children, EDHS 2016

Characteristics	Cases (N = 224) n (%)	Controls (N = 448) n (%)
Place of residence		
Urban	74 (33.0)	66 (14.7)
Rural	150 (67.0)	382 (85.3)
Region		
Tigray	16 (7.1)	42 (9.4)
Afar	11 (4.9)	42 (9.4)
Amhara	15 (6.7)	47 (10.5)
Oromia	46 (20.5)	71 (15.8)
Ethio-Somalia	18 (8.0)	54 (12.5)
Benishangul-Gumz	14 (6.2)	35 (7.8)
SNNPR	43 (19.2)	68 (15.2)
Gambela	8 (3.6)	31 (6.9)
Hareri	12 (5.4)	15 (3.3)
Addis Ababa	32 (14.3)	16 (3.6)
Dire Dawa	9 (4.1)	25 (5.6)
Sex of child		
Boys	129 (57.6)	215 (48.0)
Girls	95 (42.4)	233 (52.0)
Birth rank		
Low birth rank	141 (62.9)	218 (48.7)
High birth rank	83 (37.1)	230 (51.3)
Age of child		
Less than 6 months	46 (20.5)	43 (9.6)
6–24 months	78 (34.8)	108 (24.1)
24–59 months	100 (44.6)	297 (66.3)

#### Maternal and household characteristics

Almost half 120 (53.6%) of mothers of the cases and six out of ten 286 (63.8%) mothers of the controls didn’t attend formal education. Majority 195 (87.1%) of mothers of the cases and nine of ten mothers 420 (93.8%) of controls were not reading newspapers or magazines at all. Six out of ten 146 (65.2%) of mothers of cases and above two-third 311 (69.4%) of mothers of controls had normal BMI. Only one-third 74 (33.0%) of the cases belonged to richest category and 162 (36.2%) of the controls were poorest. Above two-third 155 (69.2%) of the households of the cases and half 243 (54.2%) of the households of controls had four and above children (Table 2).

**Table 2:** Household and maternal characteristics of under five children, EDHS 2016

Characteristics	Cases (N = 224) n (%)	Controls (N = 448) n (%)
Mother’s educational level		
No education	120 (53.6)	286 (63.8)
Primary	64 (28.6)	125 (27.9)
Secondary	25 (11.2)	22 (4.9)
Higher	15 (6.7)	15 (3.3)
Frequency of reading newspapers/magazines		
Not at all	195 (87.1)	420 (93.8)
Less than a week	17 (7.6)	25 (5.6)
At least a week	12 (5.4)	3 (0.7)
Frequency of listening radio		
Not at all	155 (69.2)	351 (78.3)
Less than a week	30 (13.4)	40 (8.9)
At least a week	39 (17.4)	57 (12.7)
Frequency of watching TV		
Not at all	158 (70.5)	372 (83.0)
Less than a week	12 (5.4)	31 (6.9)
At least a week	54 (24.1)	45 (10.0)
Mothers’ BMI		
Under weight	33 (14.7)	84 (18.8)
Normal weight	146 (65.2)	311 (69.4)
Overweight and obese	45 (20.1)	53 (11.8)
Wealth index		
Poorest	61 (27.2)	162 (36.2)
Poorer	27 (12.1)	78 (17.4)
Middle	28 (12.5)	68 (15.2)
Richer	34 (15.2)	56 (12.5)
Richest	74 (33.0)	84 (18.8)
Children ever born		
Four and above	155 (69.2)	243 (54.2)
Less than four	69 (30.8)	205 (45.8)

#### Determinants of child overweight and obesity in Ethiopia

Residence, sex and child age were significantly associated with childhood overweight and obesity in Ethiopia. The odds of overweight or obesity was 2.6 times higher among urban children than rural children (AOR = 2.6, 95% CI 1.3, 5.3). The odds of overweight were 1.5 times higher in boys compared to girls (AOR = 1.6, 95% CI 1.1, 2.2). Less than 6 months old children had three times higher odds of becoming overweight or obese (AOR = 3.4, 95% CI 2.1, 5.6) compared to 24–59 months old children. Similarly, 6–24 months old children had two times higher odds of becoming obese compared to 24–59 months old children (AOR = 2.4, 95% CI 1.6, 3.6) (Table 3).

**Table 3:** Determinants of child overweight and obesity in Ethiopia, EDHS, 2016

Characteristics	Cases, n (%)	Controls, n (%)	Odds ratio and 95% CI	
			Crude	Adjusted
Place of residence				
Urban	74 (33.0)	66 (14.7)	2.86 (1.95–4.18)	*2.63 (1.29*–5.34)*
Rural	150 (67.0)	382 (85.3)	1	*1*
Mother’s educational level				
No education	120 (53.6)	286 (63.8)	0.42 (0.20–0.89)	1.41 (0.44–4.51)
Primary	64 (28.6)	125 (27.9)	0.51 (0.24–1.11)	0.85 (0.31–2.32)
Secondary	25 (11.2)	22 (4.9)	1.14 (0.45–2.84)	1.05 (0.38–2.94)
Higher	15 (6.7)	15 (3.3)	1	1
Frequency of reading newspapers				
Not at all	195 (87.1)	420 (93.8)	0.12 (0.03–0.42)	0.26 (0.06–1.11)
Less than a week	17 (7.6)	25 (5.6)	0.17 (0.04–0.69)	0.17 (0.04–0.78)
At least a week	12 (5.4)	3 (0.7)	1	
Frequency of listening radio				
Not at all	155 (69.2)	351 (78.3)	0.65 (0.41–1.01)	
Less than a week	30 (13.4)	40 (8.9)	1.10 (0.59–2.05)	
At least a week	39 (17.4)	57 (12.7)	1	
Frequency of watching TV				
Not at all	158 (70.5)	372 (83.0)	0.35 (0.23–0.55)	0.69 (0.32–1.53)
Less than a week	12 (5.4)	31 (6.9)	0.32 (0.15–0.70)	0.42 (0.17–1.05)
At least a week	54 (24.1)	45 (10.0)	1	1
Wealth index				
Poorest	61 (27.2)	162 (36.2)	0.43 (0.28–0.66)	1.22 (0.55–2.68)
Poorer	27 (12.1)	78 (17.4)	0.39 (0.23–0.67)	1.1 (0.51–2.86)
Middle	28 (12.5)	68 (15.2)	0.47 (0.27–0.80)	1.45 (0.62–3.40)
Richer	34 (15.2)	56 (12.5)	0.67 (0.41–1.17)	2.02 (0.88–4.61)
Richest	74 (33.0)	84 (18.8)	1	1
Children ever born				
Four and above	155 (69.2)	243 (54.2)	1.90 (1.35–2.66)	1.20 (0.62–2.32)
Greater than four	69 (30.8)	205 (45.8)	1	1
Sex of child				
Boys	129 (57.6)	215 (48.0)	1.47 (1.07–2.03)	*1.56 (1.10*–2.22)*
Girls	95 (42.4)	233 (52.0)	1	*1*
BMI of mother				
Under weight	33 (14.7)	84 (18.8)	0.46 (0.26–0.82)	0.91 (0.47–1.77)
Normal weight	146 (65.2)	311 (69.4)	0.55 (0.36–0.86)	0.94 (0.54–1.62)
Overweight/obese	45 (20.1)	53 (11.8)	1	1
Birth rank				
Low birth rank (1–3)	141 (62.9)	218 (48.7)	1.79 (1.29–2.49)	1.3 (0.67–2.51)
High birth rank (> 3)	83 (37.1)	230 (51.3)	1	1
Age of child				
Less than 6 months	46 (20.5)	43 (9.6)	3.18 (1.98–5.10)	*3.40 (2.05*–5.64)*
6–24 months	78 (34.8)	108 (24.1)	2.15 (1.48–3.10)	*2.40 (1.61*–3.58)*
24–59 months	100 (44.6)	297 (66.3)	1	*1*

Italic values indicate were demographic characteristic frequency of participants

### Discussion

This study showed sex, age of the child, and place of residence were the determinants of overweight and obesity among under-five children in Ethiopia. Younger children had higher odds of becoming overweight or obese than their older counterparts. This is corroborated with studies done in Hawassa, Ethiopia [14], Gondar, Ethiopia [15], Cameroon [16], SSA [9] and Malaysia [17]. This might be explained by the fact that as age of the child increases, the chance to join kindergarten would increase and hence may attribute to increased physical activity which ultimately would lead to increased metabolic activity and energy requirements. This finding suggests that age specific nutritional counseling strategies during childhood are necessary.

Children who were living in urban areas were more affected by overweight and obesity. This is in line with the study done in Hawai [18], Peru [19] and Poland [20, 21]. This might be partly explained by differences in lifestyles, access to food supply, increased intake of energy-dense foods and rapid nutritional transition in urban areas which makes it vital for the health care providers to take the lead in practicing primary prevention and early identification of obese children in the urban communities of the country.

The relation between sex of child and overweight or obesity showed that boys were at found to be at a higher odds of overweight or obesity than girls. This is consistent with studies done in Ghana [22], Cameroon [16], China [23] and Brazil [24]. But, it was in a sharp contrast with study findings reported elsewhere [25–27] where girls were found to be affected more than the boys and where sex had no significant effect on child overweight and obesity [28]. This partly might be the result of interactions between genetic, environmental factors, metabolism, eating and physical activity behavior, and social and individual psychology [29].

### Conclusions

The finding of this study revealed that age, place of residence and sex of the children were the determinants of childhood overweight and obesity in Ethiopia. Therefore, nutrition education on feeding practices and physical activities should be boosted in the urban setting. Besides, targeted and age-specific infant and young child feeding practices are recommended to improve nutritional outcomes of children. Further study is recommended to explore other potential risk factors with the emerging problem of childhood overweight and obesity in Ethiopia.

### Limitation of the study

This secondary data analysis only focused on the specific main factors of overweight/obesity, did not include dietary intake data and feeding habits of the children.

## Data Availability

The data that support the findings of this study are available from MEASURE DHS project but restrictions apply to the availability of these data, which were used under license for the current study, and so are not publicly available. Data are however available from the MEASURE DHS project upon reasonable online request after submission of concept paper”.

## Abbreviations

BMI: body mass index
CI: confidence interval
EDHS: Ethiopia Demographic and Health Survey
CSA: Central Statistical Agency
